# Prognostic factors for the treatment of meniscus horizontal tear

**DOI:** 10.1038/s41598-022-21599-1

**Published:** 2022-10-14

**Authors:** Joon Kyu Lee, Myung Chul Lee, Joong Il Kim, Subin Lim

**Affiliations:** 1 Department of Orthopaedic Surgery, Konkuk University Medical Center, Research Institute of Medical Science, Konkuk University School of Medicine, 120-1, Neungdong-Ro, Gwangjin-Gu, Seoul, 05030 Korea; 2 Department of Orthopaedic Surgery, Seoul National University Hospital, 101 Daehak-Ro, Jongno-Gu, Seoul, 03080 Korea; 3 Department of Orthopaedic Surgery, Hallym University Kangnam Sacred Heart Hospital, 1, Singil-Ro, Yeongdeungpo-Gu, Seoul, 07741 Korea; 4 Department of Orthopaedic Surgery, Konkuk University Medical Center, 120-1, Neungdong-Ro, Gwangjin-Gu, Seoul, 05030 Korea

**Keywords:** Diseases, Medical research, Risk factors

## Abstract

Meniscus horizontal tears are usually degenerative. It could be asymptomatic and unrelated to knee symptoms. Therefore, there are controversies regarding treatment choices. The aim of this study was to evaluate factors that affect the results of non-surgical and surgical treatments for meniscus horizontal tears. We retrospectively studied 159 patients with meniscus horizontal tears with a minimum 2-year follow-up period. Patients were treated non-surgically or arthroscopically. The treatment results were dichotomized into success and failure. The factors considered were age, sex, joint line tenderness, mechanical symptoms, widest tear gap width on sagittal MRI, cartilage lesion grade, discoid meniscus, tear site, and joint alignment. Joint alignment and cartilage lesion grade were the factors that significantly influenced non-surgical treatment results. The widest tear gap width and cartilage lesion grade significantly affected arthroscopic surgery results. The mechanical symptoms did not show any significant relationship with either treatment result. In treating patients with meniscus horizontal tears, patients with varus alignment and advanced cartilage lesions should be informed of possible poor outcomes with non-surgical treatment. If the patient has a wide tear gap or minimal cartilage lesion, arthroscopic surgery would be a good treatment choice. The mechanical symptom was not an adequate factor for arthroscopic surgery.

## Introduction

Menisci are considered to act as shock absorbers, load bearers, and secondary stabilizers of the knee joint^1,2^. They are also known to have roles in joint lubrication and providing nutrition for the articular cartilage^3,4^. Meniscus tears are one of the most common injuries to the knee joint^5^. In the past, despite the important functions of the meniscus, meniscectomy was considered a treatment choice, because when the meniscus was torn, the meniscus lost its functions, and the remains were considered a cause of arthritis. However, numerous studies revealed the adverse consequences of meniscectomy. Human cadaveric studies showed increased intra-articular contact stresses after meniscectomy, leading to failure of the articular cartilage^6,7^. Since Fairbank first described radiological changes after meniscectomy^8^, plenty of reports followed with similar observations^9,10^. Although there have been arguments that the radiological changes do not always match clinical symptoms^11^, it is more likely that the severe radiological changes correlate with worse clinical outcomes^12^. Among the various types of meniscal tears, horizontal tears are usually associated with degeneration^13–15^. Because there is no disruption in the continuity of circumferential fibers in the sole horizontal tear, load-bearing, shock-absorbing functions are preserved in large part^12,16^. With relatively intact meniscal functions and already present degenerative knee condition, horizontal tear itself could be asymptomatic and not directly related to the symptom^13,14,17^. Therefore, the treatment choice for meniscus horizontal tears is controversial and requires thoughtful consideration^18^. In practice, our indications of arthroscopic surgery for horizontal meniscus tears were (1) patients with definite mechanical symptoms such as positive McMurray test, locking, clicking, and giving way^19^, who agreed to have surgery, (2) patients who failed non-surgical treatment; symptom worsened severe enough to warrant surgery after up to 6 months of non-surgical treatment.

In this study, we tried to evaluate the factors for the failure of non-surgical treatment, factors that lead to the success of arthroscopic surgery, and whether the mechanical symptom, which the authors used as the indicative factor for arthroscopic surgery, was the proper factor for the surgery. From these assessments, the authors tried to help out the decision-making of the treatment option for meniscus horizontal tear. The hypothesis was that the mechanical symptom was a critical factor in the failure of non-surgical treatment and the success of arthroscopic surgery.

## Materials and methods

**Figure 1:**
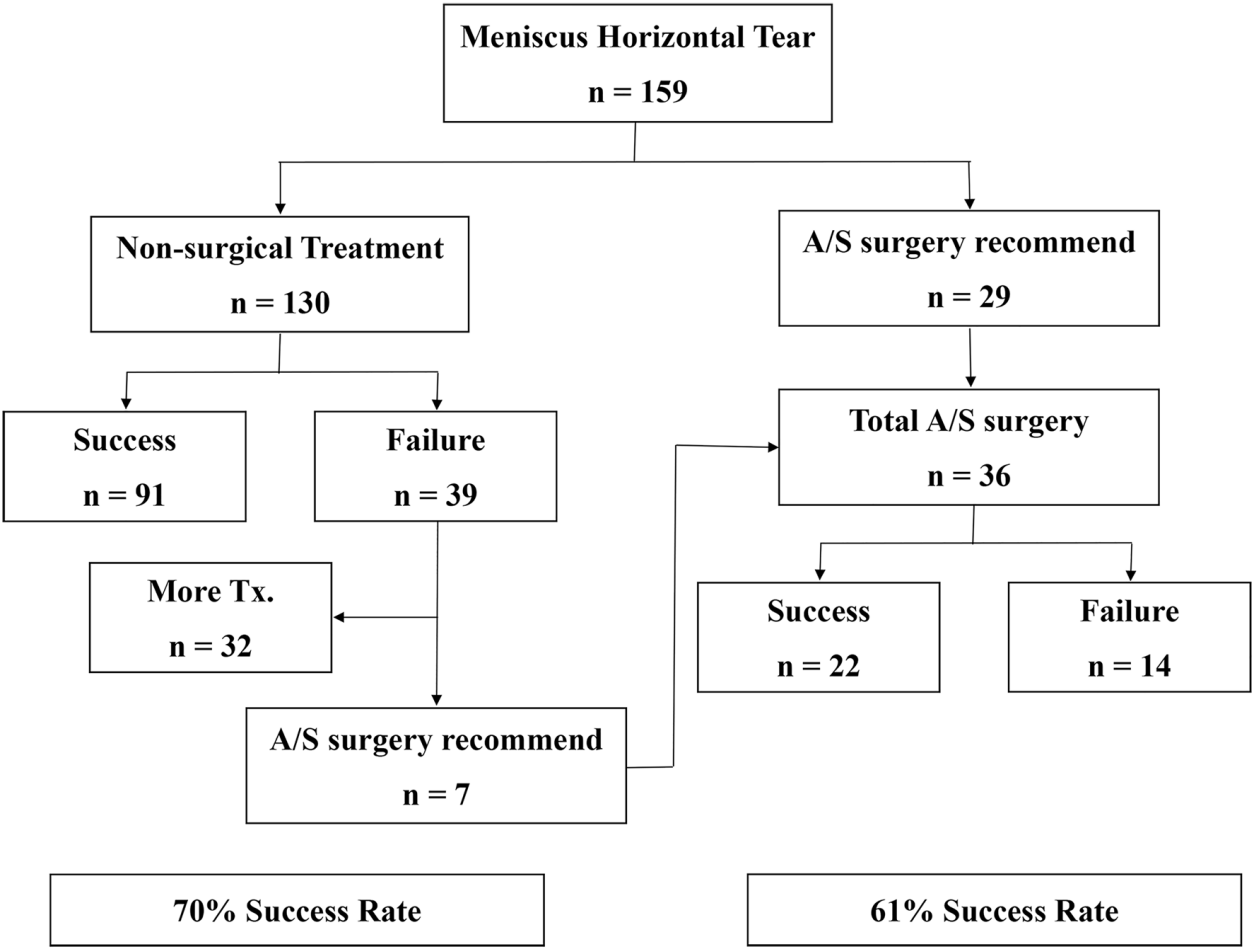
Flowchart of the study. *n* number of patients, *A/S* arthroscopic.

Between April 2008 and March 2010, two thousand, two hundred and forty-nine patients (2296 knees) visited the outpatient office with the MRI interpreted by radiologists at our institute. Patients who had meniscus horizontal tears were the subjects of this study. Exclusion criteria for the study were the patients (1) who had combined ligament injury or other types of meniscus tear such as longitudinal, radial, flap, root, and complex (853 knees), (2) who had less than a 2-year follow up (1204 knees), (3) who underwent non-arthroscopic surgery or who had operations of any kind to the knee lesion at another hospital during the 2-year follow-up (80 knees). One hundred and fifty-nine meniscus horizontal tear patients were enrolled and studied retrospectively. The average age of the patients was 54.7 years, and their ages ranged from 21 to 77 years. Forty-nine of them were male, and the other 110 were female. The average follow-up period was 26.1 months and ranged from 24 to 48 months. Data was collected by a single author (*). Initially, twenty-nine of these 159 patients were treated by arthroscopic surgery, which the indication was as described previously, and the other 130 patients were treated non-surgically. Of the 130 patients treated non-surgically, 91 patients had successful results, whereas the other 39 patients had failed. That was a 70% success rate. Among the 39 patients who had failed with non-surgical treatment, 7 patients had worsened symptoms that were sufficiently severe to warrant surgery. Therefore, a total of 36 patients underwent arthroscopic surgery. Among them, 22 patients had successful results whereas 14 patients failed, which was a 61% success rate (Fig. 1).

**Figure 2:**
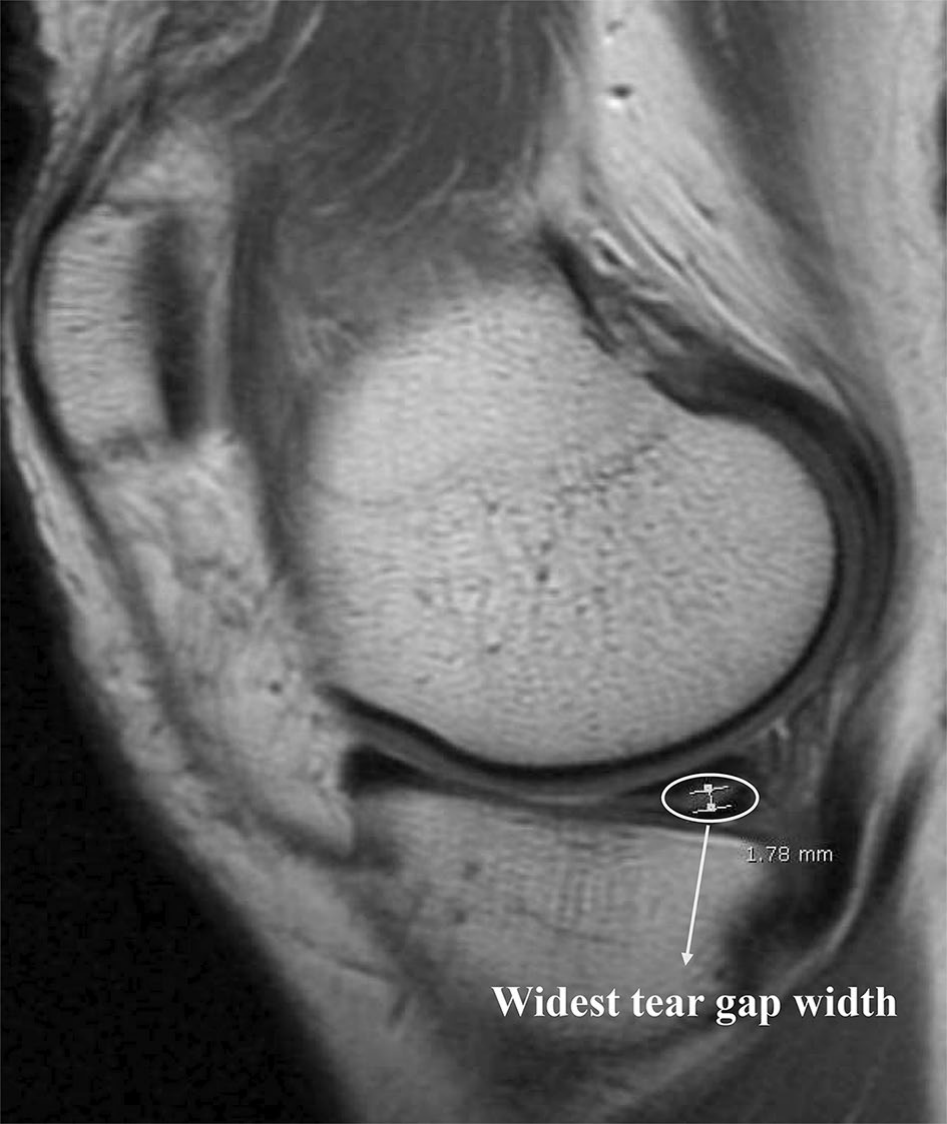
Widest tear gap width measured on the sagittal view of the MRI. Measurements were digitally analyzed via an image analyzing program (Marosis M-view 5.4, Marotech, Seoul, Korea).

Non-surgical treatment was composed of medication such as non-steroidal anti-inflammatory drugs and analgesics, recommendations of physical exercise to improve muscle strength, and some physiotherapy. All arthroscopic surgery was carried out by a single experienced surgeon (*). All of the procedures were limited resection of the unstable and displaced leaflets of a torn meniscus, mostly inferior leaflets. The success of the treatment was defined as improved symptoms and not seeking further treatment, whereas failure of the treatment was defined as no improvement or worsening in symptoms and still needing medication or physiotherapy. Factors considered for failure and success of treatments were age (divided into 3 groups; under 50, 50–60, over 60 years), sex, joint line tenderness (JLT), mechanical symptom, widest tear gap width on sagittal MRI (Fig. 2, divided into 3 groups in the evaluation of factors to the failure of non-surgical treatment; under 1.0 mm, 1.0–2.0 mm, over 2.0 mm, divided into 2 groups in the evaluation of factors to the success of arthroscopic surgery; under 2.0 mm, over 2 mm), grade of cartilage lesions (International Cartilage Repair Society (ICRS) grade, divided into 3 groups; Grade 0 or 1, Grade 2 or 3, Grade 4), discoid meniscus, meniscus tear site (divided into 3 groups; medial meniscus only, lateral meniscus only, both menisci), and joint alignment on the standing postero-anterior radiograph (tibiofemoral (TF) angle, divided into 3 groups; varus or less than 1 degree valgus, 1–4 degree valgus, over 4 degrees valgus). All the measurements on the images were digitally analyzed on a picture archiving and communication system via an image analyzing program (Marosis M-view 5.4, Marotech, Seoul, Korea). There was not a single missing data on the factors considered. JLT and mechanical symptoms were the basic physical exams and a standing postero-anterior radiograph was also the basic radiograph that we performed on every patient who visited the outpatient office. The rest of the factors could be confirmed by MRI.

This study was approved by the institutional review board at Seoul National University College of Medicine/Seoul National University Hospital (H-1009-036-331). Informed consent was obtained from all enrolled patients, either directly at follow-up office visits or over the phone. The study was performed in accordance with relevant named guidelines and regulations.

### Statistical analysis

Single variable analyses with Pearson’s chi-square tests or Fisher’s exact tests were performed to check the relationship of each factor to each topic. Then, with the factors that had meaningful relationships (p < 0.20)^20,21^, multiple logistic regression analyses were done to determine the factors that had a significant impact on the failure of non-surgical treatment or the success of arthroscopic surgery, respectively. A p-value of < 0.05 was considered significant. Statistical analysis procedures were performed using SPSS 20.0 (SPSS Inc., Chicago, IL).

## Results

### Factors to the failure of non-surgical treatment

Sex, age, JLT, TF angle, and ICRS grade were the meaningful factors in the single variable analyses (Table 1). Multiple logistic regression analysis with those factors revealed that the TF angle and ICRS grade were the only significant factors contributing to the failure of non-surgical treatment in meniscus horizontal tear (Table 2). Patients with varus alignment and advanced cartilage lesions responded poorly to non-surgical treatment.

**Table 1:** Single variable analysis results of factors to the failure of non-surgical treatment.

Factor	Success	Failure	p value^a^
Knees	91	39	
**Sex**			
Male	31	7	0.064
Female	60	32	
**Age**			
~ 50	22	2	0.101
50–60	31	19	
60 ~	38	18	
**Joint line tenderness**			
+	60	31	0.122
−	31	8	
**Mechanical symptom**			
+	28	11	0.770
−	63	28	
**TF angle**			
> 4° valgus	39	6	0.009
1°–4° valgus	29	20	
< 1° valgus	23	13	
**Tear site**			
MM	66	27	0.648
LM	18	7	
Both	7	5	
**Discoid meniscus**			
+	15	4	0.357
−	76	35	
**ICRS grade**			
0–1	62	17	0.002
2–3	28	17	
4	1	5	
**Widest tear gap width**			
~ 1 mm	34	12	0.301
1–2 mm	54	24	
2 mm ~	3	3	

^a^Pearson’s chi-square test.

*TF angle* tibiofemoral angle (joint alignment on the simple posteroanterior radiograph), *MM* medial meniscus, *LM* lateral meniscus, *ICRS* International Cartilage Repair Society.

**Table 2:** Multiple logistic regression analysis results of factors to the failure of non-surgical treatment.

Factor	Odds ratio	95% confidence interval	p value
Sex	0.928	0.315–2.738	0.893
**Age**			0.071
< 50	(Comparator)		
50–60	5.090	0.949–28.276	0.057
> 60	2.541	0.425–15.205	0.307
Joint line tenderness	1.893	0.675–5.309	0.225
**TF angle**			0.037
> 4° valgus	(Comparator)		
1°–4° valgus	4.445	1.369–14.426	0.013
< 1° valgus	3.755	1.103–12.782	0.034
**ICRS grade**			0.049
0–1	(Comparator)		
2–3	1.647	0.625–4.335	0.313
4	32.396	1.758–596.861	0.019

*TF angle* tibiofemoral angle (joint alignment on the simple posteroanterior radiograph), *ICRS* International Cartilage Repair Society.

### Factors to the success of arthroscopic surgery

Sex, widest tear gap width on sagittal MRI, and ICRS grade were meaningful factors in the single variable analyses (Table 3). Multiple logistic regression analysis with those factors revealed that the widest tear gap width and ICRS grade were the only significant factors contributing to the success of arthroscopic surgery in meniscus horizontal tear (Table 4). Patients with wide tear gaps and minimal cartilage lesions had successful results after arthroscopic surgery.

**Table 3:** Single variable analysis results of factors to the success of arthroscopic surgery.

Factor	Success	Failure	p value
Knees	22	14	
**Sex**			
Male	10	3	0.143^a^
Female	12	11	
**Age**			
~ 50	11	4	0.243^b^
50–60	8	7	
60 ~	3	3	
**Joint line tenderness**			
+	16	10	0.933^a^
−	6	4	
**Mechanical symptom**			
+	13	8	0.755^b^
−	9	5	
**TF angle**			
Valgus 4° ~	6	5	0.837^b^
1° ~ 4°	8	4	
~ 1°	8	5	
**Tear site**			
MM	15	8	0.463^b^
LM	4	5	
Both	3	1	
**Discoid meniscus**			
+	5	2	0.433^a^
−	17	12	
**ICRS grade**			
0–1	19	4	0.002^b^
2–3	2	7	
4	1	3	
**Widest tear gap width**			
~ 2 mm	10	12	0.033^a^
2 mm ~	12	2	

^a^Fisher’s exact test.

^b^Pearson’s chi-square test.

*TF angle* tibiofemoral angle (joint alignment on the simple posteroanterior radiograph), *MM* medial meniscus, *LM* lateral meniscus, *ICRS* International Cartilage Repair Society.

**Table 4:** Multiple logistic regression analysis results of factors to the success of arthroscopic surgery.

Factor	Odds ratio	95% confidence interval	p value
Sex	2.689	0.345–20.943	0.345
Widest tear gap width	8.978	1.042–77.335	0.046
**ICRS grade**			0.039
0–1	(Comparator)		
2–3	0.085	0.010–0.742	0.026
4	0.084	0.005–1.311	0.077

*ICRS* International Cartilage Repair Society.

### Mechanical symptom and the treatment results

Single variable analyses with Chi-square tests of mechanical symptom to non-surgical treatment (p value = 0.770, Table 1), and arthroscopic surgery (p value = 0.755, Table 3) showed there were no definite relationships between mechanical symptom and treatment results. The mechanical symptom was not an adequate indicative factor for arthroscopic surgery on meniscus horizontal tear.

## Discussion

The principal finding of this study was that the mechanical symptom was not an adequate factor for arthroscopic surgery on meniscus horizontal tears, whereas a wide tear gap and minimal cartilage lesions were good prognostic factors for the surgery. Additionally, meniscus horizontal tear patients with varus alignment and advanced cartilage lesions had poor results with non-surgical treatment.

In meniscus horizontal tear, since the continuity of circumferential fibers of the meniscus is intact in large part, essential functions of the meniscus are mostly preserved, and the tear itself can be relatively stable and asymptomatic^12–14,16,17^. On the other hand, the tear can cause adverse symptoms, and there seem to be occasions when arthroscopic surgeries are definitely warranted. Meniscus horizontal tears are generally considered to be degenerative tears. And these tears are also known to be highly related to cartilage degeneration^13–15^. Because there were several studies reporting inefficient results of arthroscopic surgery for meniscus degenerative tears^22,23^ and degenerative arthritis^24^, there is little consensus yet regarding the treatment choice of meniscus horizontal tear to this day. Herrlin et al. reported in their randomized controlled study that arthroscopic surgery followed by exercise therapy was not superior to the same exercise therapy alone when treating non-traumatic, degenerative medial meniscus tears in a 5-year follow-up^23^. However, in their study, among the patients who were initially treated non-surgically, one-third had complaints after 2 months of treatment and eventually received arthroscopic surgery. Yim et al. also reported in their randomized controlled trial of patients with a degenerative horizontal tear of the medial meniscus, that there were no significant differences between arthroscopic surgery and non-surgical treatment of strengthening exercises in terms of knee pain relief, knee function improvement, and patient satisfaction in a 2-year follow-up. They only included patients with daily knee pain on the medial side with mechanical symptoms^18^. However, the results of these studies showed that both arthroscopic surgery and non-surgical treatment were effective for some patients and ineffective for other patients. There must be numerous factors that affect the outcomes of these patients other than treatment modality, but these studies did not look into those factors in detail. Authors thought that because the meniscus horizontal tear is degenerative in nature, non-surgical treatment should be the first choice of treatment generally, but there should be some factors that predispose these patients to poor non-surgical treatment results and some factors that lead these patients to excellent outcomes after arthroscopic surgery. In this study, the authors focused on these factors that could affect the outcome of arthroscopic and non-surgical treatments.

Among the factors evaluated, varus alignment and advanced cartilage lesions were the factors for the poor outcome of non-surgical treatment. These two factors are related to the degenerative condition of the knee joint. The advanced cartilage lesion was also a factor in the poor outcome of arthroscopic surgery. Interestingly, patients with wider tear gap width on the sagittal view of MRI showed good results after arthroscopic surgery. Because the torn meniscus leaflet is not fixed, the tear gap width measured on MRI could be different depending on the timing of MRI taking. However, wider tear gap width could also mean greater damage to the meniscus and it could mean there was a traumatic portion of the tear, rather than only the degenerative property^25^. A wider tear gap could also lead to more symptoms and, therefore, arthroscopic surgery could have yielded better results. Surprisingly, the mechanical symptom in which the authors used as the indicative factor for arthroscopic surgery was not significantly related to the outcome of both non-surgical treatment and arthroscopic surgery. Other factors, including age, sex, joint line tenderness, combined discoid meniscus nature, and tear site also did not significantly affect the outcomes of treatments. In previous reports, some studies suggested poorer outcomes after meniscectomy in older patients^26^, on the other hand, some reported that age did not influence the outcome of meniscectomy^27^. In this study, the degenerative condition of the joint definitely affected the results of both non-surgical and surgical treatments, but age itself did not show significant relation to the results of either treatment modality. Regarding the sex to the results of meniscectomies, there were several studies that reported worse outcomes in females^28^, and there also were numerous studies that could not identify any differences^26,29^. In this study, sex did not emerge as a factor that significantly affected the outcomes of non-surgical and surgical treatments after multiple logistic regression analyses, however, single variable analysis showed that female patients had meaningfully poorer outcomes after both non-surgical treatment and arthroscopic surgery.

There are several limitations to this study. First, the results of the treatments were not analyzed in detail. No validated outcome instruments, such as well-approved clinical scores, were used. Objective results, such as radiographic changes, were not evaluated. The results were just dichotomized into success and failure only. There should be several levels of success and failure, but the authors thought that to focus on the factors, the simple classification of the results would be more beneficial. And the criteria that divided success and failure were clear that improved symptoms and not seeking further treatment at 2-year follow-up were defined as success. Further studies using validated clinical scores will be helpful to confirm our results, though. Second, the factors that were used in this study were selected rather arbitrarily. There were few studies that evaluated the results of meniscus horizontal tear and fewer studies focused on the factors in the results of meniscus horizontal tear treatment. In some studies, age and grade of osteoarthritis were used to stratify the comparison groups or to include or exclude the study subjects. In addition to general factors like age, sex, degree of joint arthritis, and knee alignment, factors such as widest tear gap width on the sagittal MRI, and existence of discoid meniscus were also evaluated as factors that affected the outcome in this study. The body mass index could be considered an important factor, however, in the usual outpatient office/clinic setting in Korea, it was hard to obtain the height and weight data of the patients, and we could not include it as one of the factors. Third, a retrospective design was used. This was because the authors had indications for arthroscopic surgery, and the patients’ agreement on surgery was required before the surgery, the prospective study could not be designed. Fourth, the exclusion criteria of the study could’ve influenced the outcome of the analysis. There were far more excluded patients than included patients. However, we wanted to focus on the meniscus horizontal tear only. Therefore, criteria 1 was necessary. With criteria 2, we do not know their treatment results. It could’ve gone either way, success or failure. There could be some patients with successful results who stopped coming to the hospital, and there could be patients who went to other hospitals and sought other treatments. In Korea, it is very easy for patients to visit several specialists in a short period of time. The expense of seeing specialists in university hospitals or private clinics is very low, so patients tend to visit several hospitals. Therefore, we had to implement criteria 2 and 3 to obtain reliable treatment results.

## Conclusion

Regardless of the factors, 70% of the meniscus horizontal tear patients were successfully treated non-surgically. Thus, the general first line of treatment for meniscus horizontal tears could be non-surgical treatment. However, when the patient has varus knee joint alignment advanced cartilage lesions, warning the patients of poor outcome would be essential. On the contrary, if the patient has wide tear gap width confirmed by MRI or minimal cartilage lesion, arthroscopic surgery would be a good treatment choice. Therefore, in deciding the treatment modality for patients with meniscus horizontal tear, thorough evaluations of the various factors should be performed case by case and not focus on the mechanical symptom alone, since the mechanical symptom is not an adequate factor for arthroscopic surgery.

## Data Availability

The datasets analyzed in this study are not publically available but are available from the corresponding author on appropriate request.
